# Isotretinoin-induced skeletal hyperostosis

**DOI:** 10.1186/2193-1801-3-698

**Published:** 2014-11-27

**Authors:** Scott W Graf, Samuel L Whittle

**Affiliations:** Department of General Medicine, Royal Adelaide Hospital, Adelaide, South Australia 5000 Australia; Rheumatology unit, The Queen Elizabeth Hospital, Woodville South, South Australia Australia

## Abstract

We describe a case of skeletal hyperostosis in a 29 year old man presenting with non-inflammatory back pain with a past history of isotretinoin therapy for acne. The development of skeletal hyperostosis, predominantly of the spine, has been reported in association with isotretinoin use and has a radiographic picture similar to diffuse idiopathic skeletal hyperostosis. The prevalence and severity of this condition appears to correlate with duration of therapy. Isotretinoin is a well-established treatment for severe acne. It is important for the rheumatologist be aware of this phenomenon when assessing young patients with musculoskeletal symptoms and evidence of radiological abnormalities.

Isotretinoin (13-cis-retinoic acid) is an oral synthetic vitamin A derivative widely used in the treatment of disorders of keratinization, in particular severe cystic acne. The development of skeletal hyperostosis has been described in association with isotretinoin therapy with radiological features similar to diffuse idiopathic skeletal hyperostosis (DISH).

A 29 year old man presented with a two year history of intermittent mild non-inflammatory pain affecting the cervical and thoracic spine. He denied any associated lower back discomfort nor any peripheral arthralgia. His past history was significant for acne for which he had been treated with isotretinoin in the past: six months of therapy nine years previously, a second 6 month course three years later and a longer three year course finishing in the year prior to his current presentation. Further history revealed no past spinal trauma, no symptoms suggestive of inflammatory bowel disease and no preceding gastrointestinal or genitourinary infection. There was no personal history of psoriasis but one first degree relative was affected.

Physical examination revealed a thoracic kyphosis with a mild painless reduction in cervical and thoracic spine range of motion. Other spinal movements were intact and painless and no sacroiliac joint tenderness was elicited. There was no peripheral synovitis, enthesitis or dactylitis and no psoriatic skin or nail changes were seen.

Blood tests revealed a normal complete blood examination, biochemistry, C-reactive protein (CRP) and erythrocyte sedimentation rate (ESR). HLA-B27 was negative. Plain radiographs showed inferior facing ossification along the C3-6 vertebral bodies (Figure 1) with flowing ossification along the mid to inferior thoracic spine (Figure 2) and small bony excrescences at the anterior aspect of the superior endplates of L2-5. Moderate enthesopathy of the iliac crest was also detected along with moderate bony osteophytes at the acetabulum bilaterally. No changes of sacroiliitis were seen. Magnetic resonance imaging (MRI) of the cervical spine was reported as unremarkable. The clinical, laboratory and radiological features were felt to be most consistent with isotretinoin-induced skeletal hyperostosis.

**Figure 1:**
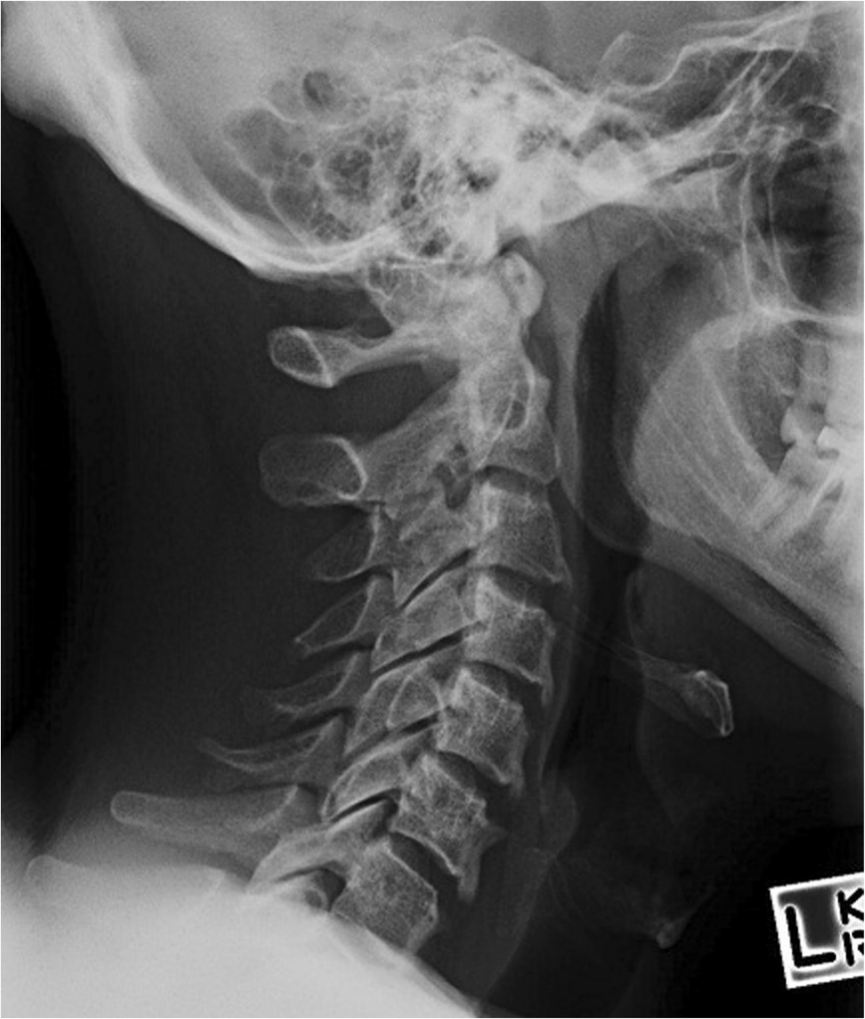
**Lateral cervical spine X-RAY showing inferiorly-facing ossification along the C3-6 vertebral bodies.**

**Figure 2:**
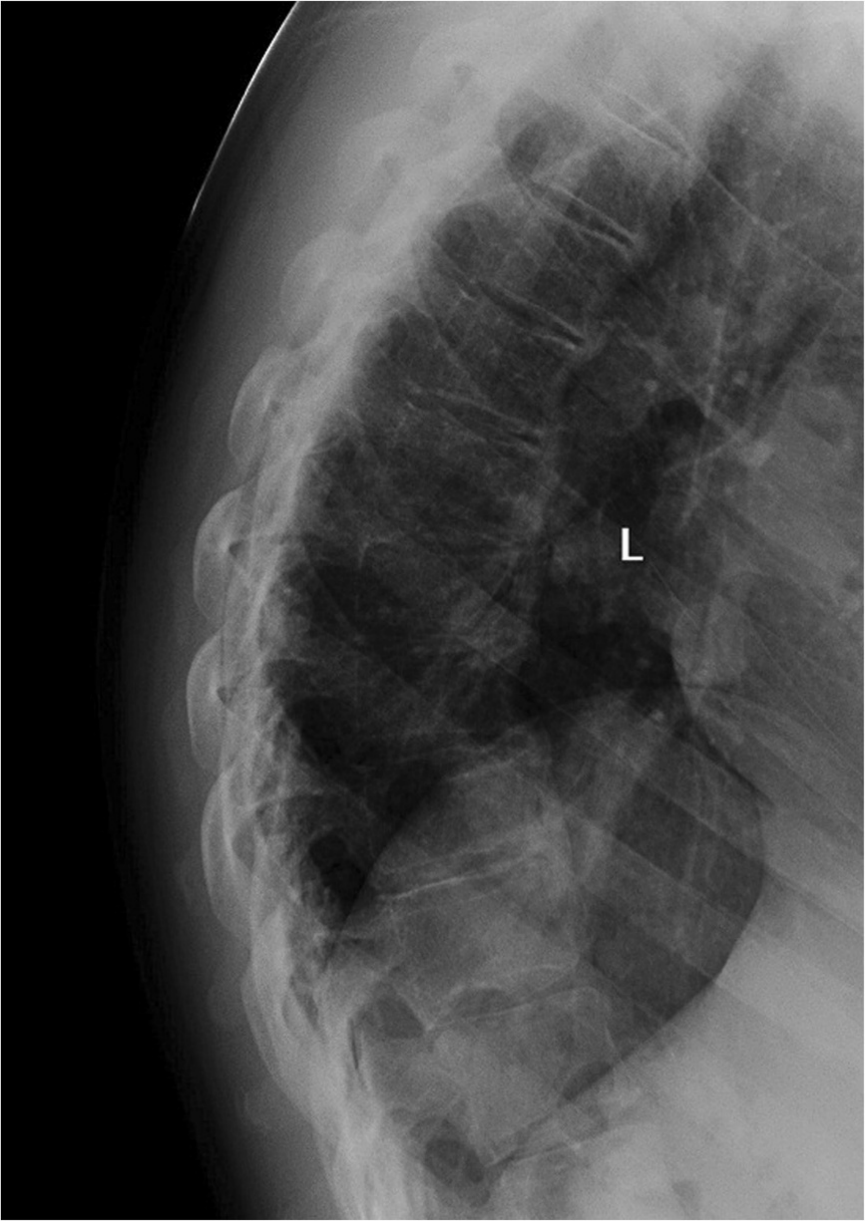
**Lateral X-RAY showing flowing ossification along mid-inferior aspects of the thoracic spine.**

Skeletal hyperostosis with isotretinoin therapy was first reported by Pittsley and Yoder in 1983 who described the development of an ossification disorder resembling DISH in 4 patients taking isotretinoin for ichthyosis (Pittsley & Yoder 1983). Further retrospective and prospective studies confirmed this association (Gerber et al. 1984; Pennes et al. 1984; Tangrea et al. 1992). Generally, higher doses of isotretinoin had been used (2 mg/kg -4.5 mg/kg) for longer periods (1–6 years) for non-acne indications.

The use of lower dose isotretinoin has also been shown to be associated with the development of hyperostosis (Tangrea et al. 1992; Kilcoyne et al. 1986). A randomised controlled trial of 139 patients with basal cell carcinoma given very low dose isotretinoin (10 mg daily) for 3 years showed significantly more progression of existing hyperostotic abnormalities and new hyperostotic involvement in the isotretinoin group compared to placebo (Tangrea et al. 1992).

The available prospective data has shown that skeletal hyperostosis can be seen as early as 6 months however the changes are subtle and of questionable clinical significance (Pennes et al. 1984; Kilcoyne et al. 1986). This raises the possibility that short courses of isotretinoin, as is currently recommended for the treatment of acne, may be unlikely to be associated with significant skeletal changes and morbidity. Follow up studies of patients treated with isotretinoin for six months or less reveal the development of only mild asymptomatic hyperostosis with no significant radiological progression seen over time (Kilcoyne et al. 1986; Ellis et al. 1988).

It is unclear whether there is a cumulative hyperostotic effect of repeated short courses of low dose isotretinoin. Ling et al. (2001) reported the development of spinal hyperostosis in 4 out of 16 patients who had received long-term and/or multiple courses of isotretinoin for acne, equivalent to three four-month courses at 1 mg/kg/day. However the study was retrospective with no baseline pre-treatment imaging and the cervical spine was not included, limiting the interpretation of these data.

The continuous long term use of isotretinoin has been shown to result in radiographic progression with hyperostosis becoming more extensive and numerous with the continuation of treatment. Spinal hyperostosis, involving the anterior vertebral bodies and anterior and posterior longitudinal ligaments, continues to progress with the development of appendicular hyperostosis appearing later, after 3 to 5 years of treatment (Ellis et al. 1988; Pennes et al. 1988).

Isotretinoin induced skeletal hyperostosis appears to be a predominately radiological syndrome with patients remaining largely asymptomatic unless the condition becomes advanced after long term therapy. Mild non-specific musculoskeletal symptoms have been reported while on therapy however these do not correlate with radiographic abnormalities. The radiological features are similar to DISH with calcification of the anterior longitudinal ligament, bony excrescences at the anterior corners of the vertebral bodies and hyperostosis at appendicular entheseal insertions. The cervical spine appears to be the predominant area involved followed by the thoracic and lumbar regions. Laboratory investigations are unremarkable (Tangrea et al. 1992; Ellis et al. 1988; Pennes et al. 1988; Carey et al. 1988).

Isotretinoin is a commonly used treatment for acne. An awareness of this phenomenon is therefore important for the rheumatologist when assessing the young patient presenting with musculoskeletal symptoms with associated radiological abnormalities.

## Consent

Informed consent was obtained from the patient for the publication of this report and any accompanying images.
